# The relationship of individual and neighbourhood deprivation with morbidity in older adults: an observational study

**DOI:** 10.1093/eurpub/ckt160

**Published:** 2013-10-21

**Authors:** Kelvin P. Jordan, Richard Hayward, Eyitope Roberts, John J. Edwards, Umesh T. Kadam

**Affiliations:** 1 Arthritis Research UK Primary Care Centre, Keele University, Keele, Staffordshire ST5 5BG, UK; 2 The Health Services Research Unit, Keele University, Keele, Staffordshire ST5 5NB, UK

## Abstract

The objective was to determine the relative association of social class and neighbourhood deprivation with primary care consultation for eight morbidities. In 18 047 survey responders aged ≥50 years, living in more deprived neighbourhoods was independently associated with new consultation for chronic obstructive pulmonary disease, ischaemic heart disease, diabetes, asthma and depression. Lower social class was associated with diabetes and chronic obstructive pulmonary disease. No such associations were found with otitis media, osteoarthritis or upper respiratory tract infection. These findings suggest a role of social environment in certain morbidities and indicate the importance of identifying and acting on neighbourhood deprivation to reduce health inequalities.

## Introduction

People in worse socioeconomic situations are often considered to have the worse health.^1^ Individual socioeconomic factors need to be placed within the context of wider social determinants, such as the neighbourhood that the individual lives in. Previous studies have examined the association of neighbourhood deprivation with morbidities such as heart disease^2^ and depression.^3^ Evidence is lacking though on the relative importance of individual- and neighbourhood-level deprivation (that is, after adjustment for each other) on development of morbidity, and whether relationships identified are consistent across all morbidities. To understand the effect of deprivation on morbidity, and whether there are public health opportunities related to tackling neighbourhood deprivation to improve health, the separate effects of individual and neighbourhood deprivation on different morbidities need to be ascertained.

The objective was to determine the relative associations of individual- and neighbourhood-level deprivation on new consultation for common morbidities in primary care in older adults.

## Methods

The study combined data from two general population surveys.^4^^,^^5^ A total of 35 620 people aged ≥50 years registered with 11 general practices in North Staffordshire, UK, were sent a postal questionnaire including general health and demographic questions and a consent form for review of medical records. Ethical approval was obtained from the North Staffordshire Local Research Ethics Committee.

The outcomes were new primary care consultation for eight common morbidities. These were ischaemic heart disease (IHD), diabetes, chronic obstructive pulmonary disease (COPD), asthma, depression, osteoarthritis/joint pain, otitis media and acute upper respiratory tract infection (URTI). The practices participate in regular training and feedback to ensure quality recording of morbidity data. A new (incident) consultation was defined by the presence of a relevant morbidity in the primary care records during the 3 years after the baseline survey with no such record during the 2 years before the survey.

The individual-level deprivation measure was social class based on self-reported current or most recent occupation.^6^ Baseline responders were allocated to one of three social class groups (i) managerial/professional; (ii) intermediate occupations/self-employed; and (iii) lower supervisory, lower technical, semi-routine or routine occupations) based on the highest social class of the individual or their spouse.

The neighbourhood measure of deprivation was the English Index of Multiple Deprivation (IMD) 2004. The IMD score is derived for geographical areas [lower level super output areas (LSOA)] consisting of a mean population of 1500. The IMD score is a weighted aggregate of data for the local area on seven domains: Income; Employment; Health Deprivation and Disability; Education, Skills and Training; Barriers to Housing and Services; Living Environment; and Crime.^7^ The LSOAs are ranked by IMD score from 1 (most deprived neighbourhood in England) to 32 482 (least deprived). Responders were grouped by quintile rank from group 1 (most deprived) to group 5 (least deprived).

Separate multilevel logistic regression models were performed with consultation (yes/no) for each morbidity in the 3 years following the baseline survey as the outcomes. People with a record of a morbidity in the 2 years before the survey were excluded from the analysis for that morbidity. Levels within the multilevel model were persons (level 1) within geographical area (LSOA, level 2). Social class and IMD group were included in each model as explanatory variables, adjusted for age, gender and general practice. Results are reported as odds ratios (OR) with 95% confidence intervals (95% CI).

To assess whether any association between individual social class and new consultation for a morbidity varied depending on the level of deprivation of a neighbourhood, cross-level interactions between individual-level social class and neighbourhood-level IMD were added to the models when both these variables were statistically significant (*P* < 0.05).

## Results

Combining both studies, 25 289 (72%) people responded to the baseline survey. Of these, 18 047 (71%) consented to medical record review and allocated to both individual and neighbourhood measures of deprivation. These 18 047 were similar to non-responders and non-consenters on age and gender. They were slightly less likely to be in the lower social class or most deprived areas than responders not consenting to record review. The 18 047 resided in 291 LSOAs (median 40 patients per LSOA; interquartile range 17–82).

The number of new consulters for each morbidity and unadjusted OR are given in the Supplementary table, with the adjusted OR shown in table 1. The highest incidence of new consultation for IHD, diabetes, COPD, asthma and depression was in the more deprived neighbourhoods. For example, consultation incidence for both IHD and COPD was over twice as high in the most deprived neighbourhoods compared with the least deprived (Supplementary table). Adjustment for social class maintained or only slightly reduced strength of associations of neighbourhood deprivation with these morbidities with the strongest associations existing for COPD and IHD. Comparing most deprived neighbourhood to least deprived, the adjusted ORs (95% CIs) were as follows: COPD 2.37 (1.67, 3.35); IHD 1.86 (1.42, 2.42); diabetes 1.51 (1.09, 2.09); depression 1.51 (1.15, 1.99) and asthma 1.49 (1.01, 2.21).

**Table 1:** Associations of new primary care consultation with individual and neighbourhood deprivation

Measure of deprivation	IHD	Diabetes	COPD	Asthma	Depression	Otitis media	OA/joint pain	URTI
	OR (95% CI)	OR (95% CI)	OR (95% CI)	OR (95% CI)	OR (95% CI)	OR (95% CI)	OR (95% CI)	OR (95% CI)
Social class								
Managerial/professional	1.00	1.00	1.00	1.00	1.00	1.00	1.00	1.00
Intermediate/self-employed	1.17 (0.97, 1.42)	1.04 (0.82, 1.34)	1.04 (0.79, 1.38)	1.28 (0.97, 1.70)	0.97 (0.79, 1.18)	0.91 (0.70, 1.19)	1.16 (1.04, 1.29)	0.97 (0.84, 1.12)
Lower supervisory/routine	1.04 (0.88, 1.24)	1.35 (1.10, 1.65)	1.42 (1.13, 1.79)	1.13 (0.88, 1.46)	1.11 (0.94, 1.32)	1.04 (0.83, 1.30)	1.06 (0.96, 1.16)	1.01 (0.89, 1.14)
Neighbourhood deprivation								
Least deprived	1.00	1.00	1.00	1.00	1.00	1.00	1.00	1.00
Second least deprived	1.18 (0.94, 1.48)	1.03 (0.80, 1.33)	1.08 (0.78, 1.50)	1.26 (0.91, 1.75)	1.06 (0.84, 1.32)	0.88 (0.67, 1.16)	0.94 (0.84, 1.05)	0.89 (0.76, 1.03)
Mid-deprived	1.29 (1.04, 1.62)	1.04 (0.80, 1.35)	1.37 (1.00, 1.87)	1.13 (0.81, 1.58)	1.30 (1.04, 1.63)	0.85 (0.64, 1.14)	1.03 (0.92, 1.17)	1.02 (0.87, 1.19)
Second most deprived	1.42 (1.11, 1.81)	1.41 (1.06, 1.87)	1.90 (1.37, 2.65)	1.12 (0.77, 1.62)	1.66 (1.30, 2.13)	0.85 (0.61, 1.19)	1.11 (0.97, 1.27)	1.04 (0.87, 1.24)
Most deprived	1.86 (1.42, 2.42)	1.51 (1.09, 2.09)	2.37 (1.67, 3.35)	1.49 (1.01, 2.21)	1.51 (1.15, 1.99)	0.75 (0.50, 1.12)	1.15 (0.99, 1.34)	0.94 (0.76, 1.17)

In those without a consultation for the morbidity in the 2 years before baseline survey, adjusted for age, gender, general practice and individual or neighbourhood deprivation.

IHD = ischaemic heart disease, COPD = chronic obstructive pulmonary disease, OA = osteoarthritis, URTI = upper respiratory tract infection.

For these five morbidities, after adjustment for neighbourhood deprivation there was an increased level of consultation for those in the lowest social class compared with the highest social class for only diabetes and COPD (diabetes OR 1.35; 95% CI 1.10, 1.65; COPD 1.42; 1.13, 1.79) and no association with social class was apparent for IHD, asthma or depression.

For diabetes and COPD, we assessed whether the association with individual-level social class varied depending on the extent of deprivation in the neighbourhood where the individual resided. This interaction was not significant, suggesting that the effect of individual social class was not moderated by the deprivation status of an area.

There was no evidence of an increasing level of consultation for osteoarthritis, URTI or for otitis media with lower social class or greater neighbourhood deprivation.

## Discussion

This study found living in more deprived neighbourhoods was associated with new consultation, most strongly for IHD and COPD but also for diabetes, asthma and depression. These associations were not explained by individual social class. Lower social class was independently associated with increased levels of new consultation for diabetes and COPD only.

There has been little previous research of the relative associations of neighbourhood and individual social class with consultation for specific morbidities but our findings reflect those from some other studies. For example, prevalence of IHD has been linked strongly to increasing neighbourhood deprivation,^8^ while Rait found a higher recorded incidence of depression in primary care in more deprived areas.^3^

The independent association of morbidity with neighbourhood deprivation is important for health care planning, as neighbourhood deprivation measures are generally more available than individual socioeconomic circumstances, which require accurate survey or Census measurements administered to large populations.

The fact that the relationship between neighbourhood deprivation and consultation is not consistent across all morbidities suggests these are morbidity-specific relationships rather than simply due to a general higher propensity to consult for any condition for those in more deprived areas. The relationship of morbidities like COPD, asthma and IHD with neighbourhood deprivation may reflect more unhealthy lifestyles (for example, increased levels of smoking, physical inactivity and poor diet) in deprived areas.^9^ Further research should examine the independent effects of neighbourhood deprivation on morbidity, controlling for these individual-level lifestyle factors, which we were unable to consider. The association of diabetes consultation with both individual and neighbourhood deprivation has added concern given deprivation has been linked to poor diabetic control and complications.^10^

There will be people in the community with a morbidity who choose not to consult, and more research is needed linking deprivation, onset of symptoms and the decision to consult. This study suggests that the level of deprivation within a person’s area of residence is associated with morbidity to a larger extent than an individual’s own socioeconomic circumstances. Such an association would require a re-examination of approaches to reducing inequalities in health, as it requires more than an intervention at the level of the individual.

## Supplementary data

Supplementary data are available at *EURPUB* online.

## Funding

The NorStOP study was supported by the Medical Research Council, UK programme grant [G9900220] and by the North Staffordshire Primary Care R&D Consortium for NHS service support costs. The KNEST study was supported by a West Midlands New Blood Research Fellowship and by the Haywood Rheumatism Research and Development Foundation, UK.

*Conflicts of interest*: None declared.

**Key points**

- There is limited evidence on the relative associations of individual social class and neighbourhood deprivation with specific morbidities.
- The influence of neighbourhood deprivation and social class appears morbidity-specific.
- Neighbourhood deprivation is as important as social class, suggesting there is a role of the social environment on development of certain morbidities.
- These findings indicate the importance of identifying and acting on the influence of neighbourhood deprivation when trying to reduce health inequalities.

## Supplementary Material

Supplementary Data
