# Supplementary material for: The relationship of individual and neighbourhood deprivation with morbidity in older adults: an observational study
Source: Eur J Public Health. 2013 Oct 21;24(3):396–8. doi: 10.1093/eurpub/ckt160 (PMC4032480; doi:10.1093/eurpub/ckt160)
Supplement: Supplementary Data [file supp_24_3_396__index.html]

The relationship of individual and neighbourhood deprivation with morbidity in older adults: an observational study — The relationship of individual and neighbourhood deprivation with morbidity in older adults: an observational study — Supplementary Data 

# The relationship of individual and neighbourhood deprivation with morbidity in older adults: an observational study

## Supplementary Data

files

**Files in this Data Supplement:**

- Supplementary Data - docx file
