# Supplementary material for: The relationship of individual and neighbourhood deprivation with morbidity in older adults: an observational study
Source: Eur J Public Health. 2013 Oct 21;24(3):396–8. doi: 10.1093/eurpub/ckt160 (PMC4032480; doi:10.1093/eurpub/ckt160)
Supplement: Supplementary Data [file supp_ckt160_ejph-2013-02-sr-0127-File002.docx]

Supplementary tables. Consultation incidences over 3 years and unadjusted associations with individual and neighbourhood deprivation

|  |  | IHD |  |  | Diabetes |  |  | COPD |  |
| --- | --- | --- | --- | --- | --- | --- | --- | --- | --- |
|  | *n*^a^ | Consult^b^  *n* (%) | OR (95% CI)^c^ | *n*^a^ | Consult^b^  *n* (%) | OR (95% CI)^c^ | *n*^a^ | Consult^b^  *n* (%) | OR (95% CI)^c^ |
| Total | 16592 | 966 (6) |  | 17015 | 629 (4) |  | 17285 | 536 (3) |  |
| Social class |  |  |  |  |  |  |  |  |  |
| managerial/professional | 4888 | 237 (5) | 1.00 | 5033 | 152 (3) | 1.00 | 5147 | 109 (2) | 1.00 |
| intermediate/self employed | 3749 | 235 (6) | 1.27 (1.05, 1.54) | 3838 | 123 (3) | 1.06 (0.83, 1.35) | 3888 | 100 (3) | 1.18 (0.89, 1.56) |
| lower supervisory/ routine | 7955 | 494 (6) | 1.22 (1.03, 1.44) | 8144 | 354 (4) | 1.43 (1.17, 1.74) | 8250 | 327 (4) | 1.77 (1.41, 2.23) |
| Neighbourhood deprivation | | |  |  |  |  |  |  |  |
| least deprived | 3291 | 139 (4) | 1.00 | 3407 | 113 (3) | 1.00 | 3440 | 66 (2) | 1.00 |
| 2^nd^ least deprived | 4304 | 208 (5) | 1.15 (0.88, 1.51) | 4412 | 147 (3) | 1.00 (0.75, 1.34) | 4491 | 90 (2) | 1.04 (0.76, 1.44) |
| mid deprived | 3955 | 237 (6) | 1.43 (1.10, 1.86) | 4053 | 141 (3) | 1.04 (0.77, 1.40) | 4116 | 122 (3) | 1.56 (1.15, 2.12) |
| 2nd most deprived | 2737 | 185 (7) | 1.67 (1.27, 2.20) | 2805 | 127 (5) | 1.37 (1.01, 1.85) | 2870 | 126 (4) | 2.35 (1.74, 3.17) |
| most deprived | 2305 | 197 (9) | 2.15 (1.64, 2.82) | 2338 | 101 (4) | 1.32 (0.97, 1.80) | 2368 | 132 (6) | 3.02 (2.24, 4.08) |

|  |  | Asthma |  | Depression | | | Otitis Media | | |
| --- | --- | --- | --- | --- | --- | --- | --- | --- | --- |
|  | *n*^a^ | Consult^b^  *n* (%) | OR (95% CI)^c^ | *n*^a^ | Consult^b^  *n* (%) | OR (95% CI)^c^ | *n*^a^ | Consult^b^  *n* (%) | OR (95% CI)^c^ |
| Total | 17072 | 405 (2) |  | 16963 | 885 (5) |  | 17631 | 459 (3) |  |
| Social class |  |  |  |  |  |  |  |  |  |
| managerial/professional | 5008 | 100 (2) | 1.00 | 4981 | 234 (5) | 1.00 | 5156 | 144 (3) | 1.00 |
| intermediate/self employed | 3827 | 104 (3) | 1.35 (1.02, 1.79) | 3810 | 183 (5) | 1.01 (0.83, 1.23) | 3959 | 95 (2) | 0.86 (0.66, 1.12) |
| lower supervisory/ routine | 8237 | 201 (2) | 1.20 (0.94, 1.54) | 8172 | 468 (6) | 1.20 (1.02, 1.41) | 8516 | 220 (3) | 0.93 (0.75, 1.15) |
| Neighbourhood deprivation | |  |  |  |  |  |  |  |  |
| least deprived | 3368 | 62 (2) | 1.00 | 3328 | 138 (4) | 1.00 | 3451 | 104 (3) | 1.00 |
| 2^nd^ least deprived | 4367 | 99 (2) | 1.22 (0.86, 1.73) | 4324 | 203 (5) | 1.13 (0.88, 1.45) | 4507 | 126 (3) | 0.92 (0.69, 1.22) |
| mid deprived | 4080 | 98 (2) | 1.28 (0.90, 1.81) | 4043 | 216 (5) | 1.30 (1.01, 1.66) | 4220 | 110 (3) | 0.86 (0.64, 1.15) |
| 2nd most deprived | 2858 | 71 (2) | 1.33 (0.92, 1.93) | 2865 | 180 (6) | 1.55 (1.20, 2.00) | 2968 | 73 (2) | 0.81 (0.59, 1.11) |
| most deprived | 2399 | 75 (3) | 1.68 (1.17, 2.42) | 2403 | 148 (6) | 1.53 (1.17, 1.99) | 2485 | 46 (2) | 0.60 (0.42, 0.87) |

|  | OA / joint pain | | |  | URTI |  |
| --- | --- | --- | --- | --- | --- | --- |
|  | *n*^a^ | Consult^b^  *n* (%) | OR (95% CI)^c^ | *n*^a^ | Consult^b^  *n* (%) | OR (95% CI)^c^ |
| Total | 13391 | 3705 (28) |  | 16474 | 1768 (11) |  |
| Social class |  |  |  |  |  |  |
| managerial/professional | 4041 | 1048 (26) | 1.00 | 4833 | 521 (11) | 1.00 |
| intermediate/self employed | 2999 | 883 (29) | 1.19 (1.07, 1.32) | 3698 | 389 (11) | 0.97 (0.84, 1.12) |
| lower supervisory/ routine | 6351 | 1774 (28) | 1.10 (1.01, 1.21) | 7943 | 858 (11) | 1.00 (0.89, 1.13) |
| Neighbourhood deprivation | | |  |  |  |  |
| least deprived | 2683 | 733 (27) | 1.00 | 3220 | 360 (11) | 1.00 |
| 2^nd^ least deprived | 3459 | 902 (26) | 0.94 (0.83, 1.06) | 4203 | 440 (10) | 0.93 (0.74, 1.17) |
| mid deprived | 3179 | 893 (28) | 1.04 (0.92, 1.18) | 3940 | 444 (11) | 0.99 (0.79, 1.25) |
| 2nd most deprived | 2221 | 653 (29) | 1.10 (0.97, 1.26) | 2762 | 314 (11) | 1.01 (0.79, 1.28) |
| most deprived | 1849 | 524 (28) | 1.05 (0.92, 1.21) | 2349 | 210 (9) | 0.75 (0.59, 0.97) |

^a^ total number without a consultation for the morbidity in the 2 years before baseline survey, ^b^ consulted for the morbidity in the 3 years after baseline survey ^c^ unadjusted

IHD = ischaemic heart disease, COPD = chronic obstructive pulmonary disease, OA = osteoarthritis, URTI = upper respiratory tract infection
